# Interaction of Soil Texture and Irrigation Level Improves Mesophyll Conductance Estimation

**DOI:** 10.3390/plants14243784

**Published:** 2025-12-12

**Authors:** Lu Lin, Pengpeng Wang, Zhenxu Liang, Mingde Sun, Yang Zhao, Hongning Wang, Kai Zhu, Lu Yu, Songzhong Liu, Zhiqiang Li

**Affiliations:** 1 College of Horticulture, Shanxi Agricultural University, Taiyuan 030031, China; linluyyxy@sxau.edu.cn (L.L.); 18706933239@163.com (P.W.);; 2 Institute of Forestry & Pomology, Beijing Academy of Agriculture & Forestry Sciences, Beijing 100093, China; 3 Liaocheng Academy of Agricultural Sciences, Liaocheng 252000, China; 4 Institute of Pomology, Shanxi Agricultural University, Taiyuan 030031, China; 5 College of Resources and Environment, Shanxi Agricultural University, Jinzhong 030801, China

**Keywords:** mesophyll limitation, photosynthesis, biochemical model, clay soil, sandy soil, loam soil, irrigation level, interactive effects, fruit trees

## Abstract

Combining leaf gas exchange with chlorophyll fluorescence, this study quantified the effects of soil water content (*SWC*) on mesophyll conductance (*g*_m_) and biochemical parameters in 8-year-old pear trees across three soil textures [clay (CS), sandy (SS), loam (LS)], each subjected to three irrigation levels (100%FI, 75%FI, 50%FI). Results showed that *SWC* differed significantly, with CS > LS > SS, and that the difference in *SWC* in loam soil was the most obvious among different irrigation levels. The leaf water content (*LWC*) of SS was higher than that of LS and CS, and SS_50%FI_ showed 7.53% and 13.30% greater *LWC* compared to LS_50%FI_ and CS_50%FI_, respectively. Specific leaf area (*SLA*) peaked at CS_75%FI_ and SS_100%FI_. Soil texture and irrigation level had significant interactive effects on *g*_m_, the product of light absorption coefficient and light energy partitioning ratio (*α*·*β*), leaf apparent CO_2_ compensation point, dark respiration rate under light, and photosynthetic biochemical parameters. Differences in the values of *α*·*β* among the nine treatments were significant and the maximum values in the three soil textures were 0.660 (LS_75%FI_), 0.366 (SS_100%FI_) and 0.462 (CS_50%FI_), respectively. The most sensitive treatment of *g*_m_, responding to photosynthetically active radiation (*PAR*), was SS_100%FI_ and the maximal *g*_m_ under saturated *PAR* reached 0.271 molCO_2_·m^−2^·s^−1^, increasing 2.2-fold and 8.8-fold compared to that of SS_75%FI_ and SS_50%FI,_ respectively. An underestimation of 26.4% to an overestimation of 30.3% for *g*_m_ and an underestimation of 28.8% to an overestimation of 15.5% were observed for biochemical parameters if the empirical value (0.425) of *α*·*β* was adopted. Our findings indicated that the maximum leaf *g*_m_ could be obtained at 75%FI for loam soil, 100% FI for sandy soil, and 50% FI for clay soil, respectively.

## 1. Introduction

The mesophyll conductance (*g*_m_) of plant leaves is defined as the conductance of CO_2_ diffusing from the substomatal cavity of the leaf into the chloroplast. It numerically equals the reciprocal of the mesophyll cell resistance to CO_2_ diffusion [1]. Net photosynthetic rate (*A*_n_), most frequently adopted by existing studies on leaf photosynthesis of horticultural plants (such as fruit trees), is calculated as the difference between ambient atmospheric CO_2_ concentration and the concentration after CO_2_ is absorbed by the leaf [2,3]. Importantly, leaf photosynthetic capacity cannot be precisely evaluated using *A*_n_ due to variations in concentration, regardless of whether CO_2_ enters the stomata [4]. Leaf *g*_m_ depends on the CO_2_ concentration at the carboxylation site in the chloroplast (*C*_c_) by directly affecting mesophyll intracellular CO_2_ diffusion. Thus, *g*_m_ is significantly related to the photosynthetic efficiency and potential of leaves [5,6]. Obviously, *g*_m_ plays a crucial role in evaluating the leaf photosynthetic capacity of fruit trees accurately.

Numerous published reports on the photosynthetic physiology of fruit trees have neglected leaf *g*_m_ by assuming that intercellular CO_2_ concentration (*C*_i_) equals *C*_c_ [7,8,9,10]. However, this will lead to system deviations in the parameters estimated from the photosynthesis biochemical model if *g*_m_ is not taken into account [11]. Some studies considering *g*_m_ substituted the ratio of *A*_n_ to *C*_i_ for the equivalent of *g*_m_ [12,13]. There are also some studies that adopted empirical values for important parameters used for calculating *g*_m_, such as the ratio of incident light absorbed by the leaf (*α*), where the ratios of light energy partitioned between photosystem I (PSI) and photosystem II (PSII) (*β*) are taken as 0.85 and 0.5, respectively [14,15]. Nevertheless, a growing disagreement among scholars has arisen regarding the use of empirical values for *α* and *β*. Theoretically, *α* and *β* are related to calibration methods (such as *A*_n_/*C*_i_ or *A*_n_/*PPFD* curves), species, and environmental conditions [16], but experimental evidence remains scarce to date.

Soil water condition is a crucial limiting factor impacting leaf photosynthesis in plants [17,18,19]. Soil water content (*SWC*) determines the efficiency of photosynthetic carbon assimilation by regulating stomatal (*g*_s_) and mesophyll (*g*_m_) conductance. Studies have shown that when the soil relative water content fell below a critical threshold (such as 52% for cassava), *SWC* became the primary limiting factor for *g*_s_. Reduction in leaf photosynthetic productivity fundamentally results from the decreased *C*_c_ induced by the decline of stomatal and mesophyll conductance of leaves when plants are suffering from water stress [17,20,21]. The variation in *g*_m_ may be related to leaf structural characteristics (such as mesophyll thickness and chloroplast distribution), which need to be verified through anatomical measurements in future studies. Soil texture is a crucial attribute of soil, closely related to its moisture retention and thus its water content. Soil texture affects soil moisture availability by regulating soil pore structure (determining water retention and permeability) and hydraulic properties (such as water holding capacity and water conductivity), thereby affecting crop growth and water management strategies in production [22,23,24]. Sandy soil has a high proportion of macropores but a low water-holding capacity. Clay soil has abundant fine pores and a strong water-holding capacity, while loam soil has both good water retention and permeability. Soil texture is also a key regulatory factor in determining water availability in the soil. Its influence is not only reflected in *SWC*, but also in the water availability environment it shapes—the effect of changes in *SWC* on *g*_m_. Previous studies have demonstrated that drought stress can negatively impact plant growth [25,26,27]. However, there is still a lack of systematic research on how soil texture regulates *g*_m_ by altering *SWC*. In theory, soil texture may have a significant impact on *g*_m_ by affecting *SWC*, nutrient availability, gas exchange, and other soil processes [28]. However, up to now, little attention has been paid to the effects of soil water content under different types of soil texture on leaf *g*_m_, especially the relevant key parameters. Previous studies on *g*_m_ quantification under drought or salt stress have mainly focused on herbaceous plants and ornamental trees (*Cotinus coggygria*) [29,30,31], while few have examined fruit trees—especially pear trees—which have significant economic value. In addition, most studies ignore the interaction between soil texture and water content and rarely quantify changes in *α*·*β* across different soil conditions. This study focused on pear trees to explore the combined effects of soil texture and irrigation levels on *g*_m_ and photosynthetic biochemical parameters, and to verify the limitations of empirical *α*·*β* values in photosynthetic research of fruit trees.

At present, the pear cultivation area in China is 1.0003 million hectares (accounting for 68% of the world’s total cultivation area), and is mainly distributed in the Bohai Bay, the Loess Plateau, and the middle and lower reaches of the Yangtze River. These production areas involve terrain types such as plains, mountains, hilly areas, and river beaches, so the soil textures are extremely complex. At the same time, its photosynthetic capacity is highly sensitive to soil water content, which directly affects fruit yield and quality. Therefore, it is an urgent problem to study and establish high-quality, high-yield, and water-saving technologies for pear trees, especially those suitable for different site conditions (e.g., different soil textures). In addition, the pear tree is a typical temperate woody fruit tree with perennial, deep roots and seasonal physiological characteristics. Its leaf structure and photosynthetic apparatus are similar to those of other important Rosaceae fruit trees (such as apples and peaches) and many woody crops. Therefore, the regulation of the interaction between soil textures and water management on mesophyll conductance revealed in pear trees may also be applicable to other fruit tree species with similar growth habits and leaf structure.

Accordingly, the present study was conducted to quantify the effects of soil water content (achieved through application of different irrigation levels) on leaf *g*_m_ and relevant parameters of pear trees across various soil textures, based on measurements of gas exchange and chlorophyll fluorescence. It can serve as a theoretical basis for the accurate prediction of plant photosynthetic capacity responses to different soil water conditions and provide guidance for water-saving irrigation in orchards covering a wider range of soil conditions.

## 2. Results

### 2.1. Changes in Dynamics of Soil Water Content

**Figure 1:**
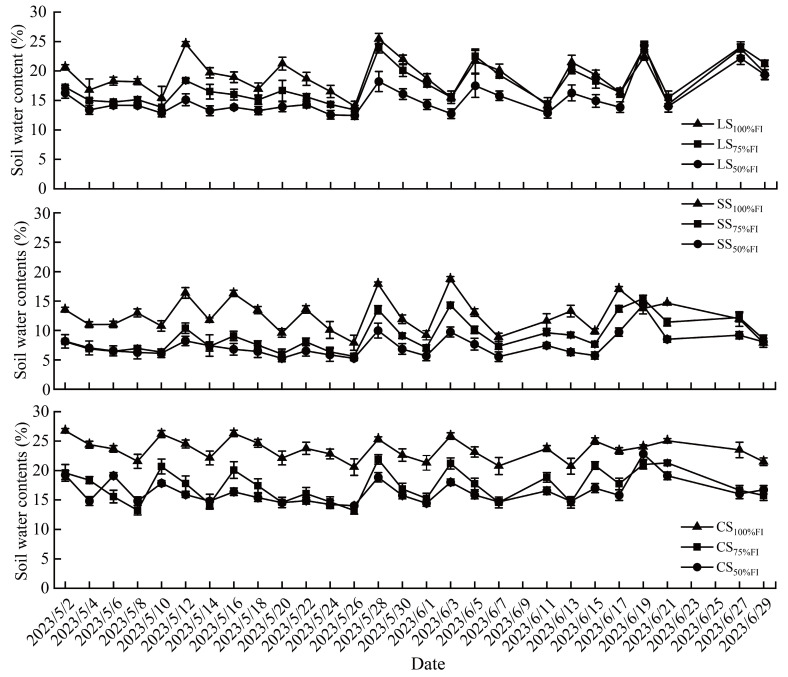
Changes in soil water content in the root layer of pear trees (mean value of 0~40 cm below the ground surface) of 9 treatments during the experimental period (May and June 2023).

Soil water contents (*SWC*) of the three soil textures differed significantly, even when the same irrigation levels were applied (Figure 1). The averaged *SWC* of CS_100%FI_, LS_100%FI_, and SS_100%FI_ during the experimental period (May and June in 2023) were 25.53%, 19.07% and 12.52%, respectively. The differences in *SWC* among the three soil textures decreased with the reduction of irrigation level; however, the averaged value of *SWC* still manifested as CS_75%FI_ > LS_75%FI_ > SS_75%FI_. When the irrigation level was reduced to 50% FI, the averaged SWC of the three soil textures in sequence (from high to low) was 16.37% (CS_50%FI_), 15.26% (LS_50%FI_), and 7.43% (SS_50%FI_), respectively.

For the same soil textures, *SWC* decreased as irrigation levels decreased (Figure 1). The difference of *SWC* in loam soil (LS) was the most significant among different irrigation levels, and the *SWC* ranges in LS_100%FI_, LS_75%FI,_ and LS_50%FI_ were 14.03~25.45%, 13.39~24.08% and 12.44~24.33%, respectively. The *SWC* ranges of three irrigation levels in clay soil (CS) (CS_100%FI_, CS_75%FI,_ and CS_50%FI_) during the experimental period were 20.56~26.77%, 13.17~21.87% and 14.02~22.82%, respectively. For sandy soil (SS), the *SWC* ranges of the three irrigation levels were 7.91~18.71% (SS_100%FI_), 5.62~15.39% (SS_75%FI_), and 5.25~14.30% (SS_50%FI_), respectively.

### 2.2. Changes in Leaf Water Content and Specific Leaf Area

**Figure 2:**
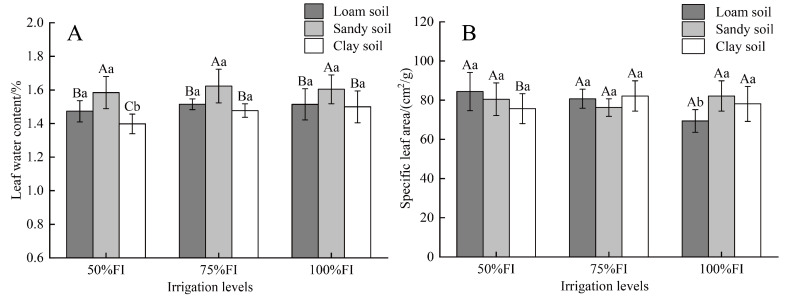
Differences in leaf water content (*LWC*) (**A**) and specific leaf area (*SLA*) (**B**) among 9 treatments. Note: Different capital letters indicate that there were significant differences between different soil textures under the same irrigation level (*p* < 0.05). Different lowercase letters indicate significant differences in the same irrigation level across different soil textures (*p* < 0.05).

As shown in Figure 2, there were significant differences in the leaf water content (*LWC*) and specific leaf area (*SLA*) of pear trees under different treatments. For an irrigation level of 50%FI, the *LWC* in SS was higher than that in LS and CS, increasing by 7.53% and 13.30%, respectively. This suggests that under low *SWC*, pear trees in sandy soil exhibit a superior ability to maintain leaf hydration. For an irrigation level of 75%FI, the *LWC* of LS and CS was significantly lower than that of SS, with reductions of 7.17% and 9.92%, respectively. When the irrigation level was set to 100%FI, the *LWC* among the three soil textures was ranked as follows: SS > LS > CS. The *LWC* varied significantly among different irrigation levels within the same soil textures. The peak *LWC* in LS and SS was observed in the 75%FI treatment. The *LWC* of LS was highest under the LS_75%FI_ treatment, with no significant difference between the 100%FI and 75%FI treatments, whereas the 50%FI treatment significantly decreased it (*p* < 0.05). For SS, the decreasing order was SS_75%FI_ > SS_100%FI_> SS_50%FI_. In CS, the *LWC* increased with increasing irrigation, following the hierarchy: CS_100%FI_ > CS_75%FI_ > CS_50%FI_ (Figure 2A).

Different soil textures significantly influenced the *SLA* under the same irrigation levels (Figure 2B). At an irrigation level of 50% FI, the *SLA* of LS and SS were significantly higher than that of CS, by 21.63% and 16.33%, respectively. As the irrigation level increased to 75% FI, the *SLA* values for the different soil textures were ranked as follows: CS_75%FI_ > LS_75%FI_ > SS_75%FI_. When an irrigation level of 100%FI was applied, the *SLA* of SS surpassed that of both CS and LS. The *SLA* varied significantly among different irrigation levels within the same soil textures. The *SLA* of LS decreased as the irrigation levels increased (Figure 2B). The *SLA* of sandy soil was highest under the SS_100%FI_ and lowest under the SS_75%FI_. In the case of clay soil, the *SLA* at CS_100%FI_ and CS_75%FI_ did not differ significantly; however, both were significantly higher than that at CS_50%FI_, with increments of 12.57% and 18.61%, respectively.

### 2.3. Responses of g_m_ and the Related Parameters to Interactions of Soil Texture and Irrigation Level

Soil texture and irrigation level significantly influenced leaf CO_2_ diffusion conductance and related parameters (*p* < 0.05) according to two-way ANOVA (Table 1). Significant interactive effects of soil textures and irrigation levels were observed on *g*_m_, *α·β*, *C*_i_*, *R*_d_, *V*_cmax_, *J*_max_ and *V*_tpu_ (*p* < 0.001, *p* = 0.0159, *p* < 0.001, *p* < 0.001, *p* < 0.001, *p* < 0.001, and *p* < 0.001, respectively). Irrigation levels significantly affected *g*_m_, *α·β*, *C*_i_*, and *R*_d_, while soil textures significantly affected *g*_m_, *C*_i_*, *R*_d_, *V*_cmax,_ and *J*_max_. These interactive effects suggest that both soil textures and irrigation levels jointly regulate the CO_2_ diffusion pathway and biochemical processes within the leaf, likely through modifications to mesophyll anatomy and chloroplast organization.

### 2.4. The Difference of α·β Value Under Different SWC

**Figure 3:**
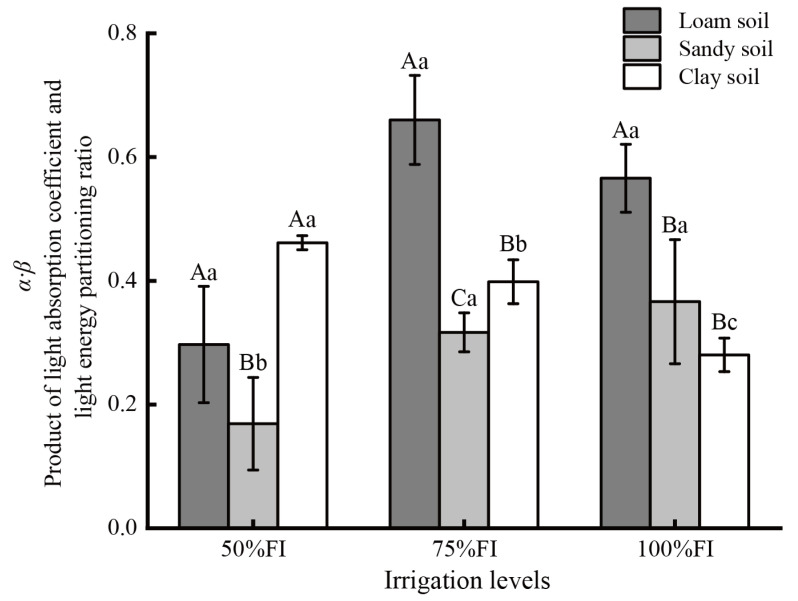
Differences in the product of the light absorption coefficient and the light energy partitioning ratio (*α·β*) among 9 treatments. Note: Different capital letters indicate that there were significant differences between different soil textures at the same irrigation level (*p* < 0.05). Different lowercase letters indicate significant differences in the same irrigation level across different soil textures (*p* < 0.05).

The product of the light absorption coefficient and the light energy partitioning ratio (*α·β*) was significantly influenced by soil water conditions. For an irrigation level of 50%FI, the *α·β* value of CS was significantly higher than that of LS and SS (increased by 55.4% and 1.73-fold, respectively) (Figure 3). As the irrigation level increased to 75%FI, the *α·β* values for the three soil textures differed significantly, with LS 65.7% higher than CS and CS 25.7% higher than SS. When the irrigation level was set to 100%FI, the *α·β* values between SS and CS were not significantly different. In contrast, both were significantly lower than LS (decreased by 35.3% and 50.4%, respectively).

The divergences in *α·β* values across the three soil textures also differed with irrigation treatments (Figure 3). For LS, the highest *α·β* value of leaves responded to 75%FI, and the difference among the three irrigation levels was LS_75%FI_ > LS_100%FI_ > LS_50%FI_. The *α·β* values of leaves in SS increased with increasing irrigation levels, and the *α·β* values for SS_50%FI_, SS_75%FI_, and SS_100%FI_ were 0.169, 0.317, and 0.366, respectively. In contrast, the *α·β* values of leaves in CS decreased with increasing irrigation levels, and the differences among the three irrigation levels were significant, with the maximum at CS_50%FI_, the medium at CS_75%FI_, and the minimum at CS_100%FI_. The significant changes in the *α·β* value indicated that the absorption and partitioning of light energy by the photosynthetic apparatus were not fixed, but dynamically regulated by the plant’s water status, which was a direct consequence of the *SWC*.

### 2.5. The Differences of C_i_* and R_d_ Under Different SWC

**Figure 4:**
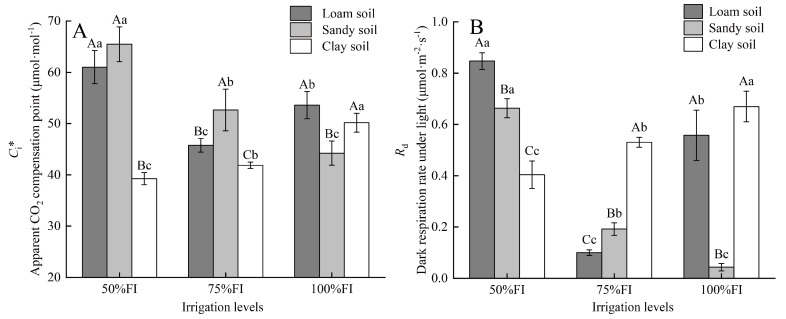
Differences in apparent CO_2_ compensation point (*C*_i_*) (**A**) and dark respiration rate under light (*R*_d_) (**B**) among 9 treatments. Note: Different capital letters indicate that there were significant differences between different soil textures at the same irrigation level (*p* < 0.05). Different lowercase letters indicate significant differences in the same irrigation level across different soil textures (*p* < 0.05).

The leaf apparent CO_2_ compensation point (*C*_i_*) and dark respiration rate under light (*R*_d_) varied significantly with *SWC* conditions (Figure 4). For the 50%FI irrigation level, leaf *C*_i_* of SS and LS treatment (65.5 and 61.0 μmol·mol^−1^, respectively) was significantly higher than that of CS (39.3 μmol·mol^−1^). When it came to the irrigation level of 75%FI, the *C*_i_* of leaves in SS (52.7 μmol·mol^−1^) was significantly higher than that in LS (45.8 μmol·mol^−1^), and the latter was significantly higher than that in CS (41.9 μmol·mol^−1^). At an irrigation level of 100%FI, the *C*_i_* of leaves in SS (44.2 μmol·mol^−1^) was remarkably lower than that of LS (53.6 μmol·mol^−1^) and CS (50.2 μmol·mol^−1^). The *C*_i_* across different irrigation levels under the same soil texture conditions also showed significant differences (*p* < 0.05). The lowest leaf *C*_i_* in LS responded to the medium irrigation level (LS_75%FI_), which was 14.6% and 25.0% lower than LS_100%FI_ and LS_50%FI_, respectively. The *C*_i_* of leaves in SS decreased monotonically as the irrigation levels increased, and that of SS_100%FI_ was 16.0% and 32.4% lower than that of SS_75%FI_ and SS_50%FI_, respectively. On the contrary, the *C*_i_* of leaves in CS increased progressively with the added irrigation levels, and the leaf *C*_i_* of CS_100%FI_ was 19.8% and 27.8% higher than that of CS_75%FI_ and CS_50%FI_, respectively.

The minimum (0.10 μmol·m^−2^·s^−1^), medium (0.56 μmol·m^−2^·s^−1^), and maximum (0.85 μmol·m^−2^·s^−1^) of leaf *R*_d_ in LS responded to irrigation levels of 75% FI, 100%FI, and 50%FI, respectively (Figure 4). The *R*_d_ value of leaves in SS decreased constantly with the increase in irrigation levels, and those of SS_50%FI_, SS_75%FI_, and SS_100%FI_ were 0.66, 0.19, and 0.04 μmol·m^−2^·s^−1^, respectively. Conversely, in CS, leaf *R*_d_ increased in proportion to the increments in irrigation level, with 0.40 μmol·m^−2^·s^−1^ of CS_50%FI_, 0.53 μmol·m^−2^·s^−1^ of CS_75%FI_, and 0.67 μmol·m^−2^·s^−1^ of CS_100%FI_. *R*_d_ values showed more significant differences among the three soil textures at the same irrigation levels. The leaf *R*_d_ in CS_100%FI_ and LS_100%FI_ were 14.5-fold and 11.9-fold higher, respectively, compared to SS_100%FI_, which was a significant difference. Under an irrigation level of 75%FI, the leaf *R*_d_ in CS was significantly higher than that in SS (increased by 1.8-fold), while that in SS was significantly higher than in LS (increased by 92.2%). When the irrigation level decreased to 50% FI, the highest and lowest leaf *R*_d_, respectively, responded to LS (27.7% higher than SS_50%FI_, *p* < 0.05) and CS (39.1% lower than SS_50%FI_).

### 2.6. Responsive Characteristics of g_m_ to PAR Under Different SWC

**Figure 5:**
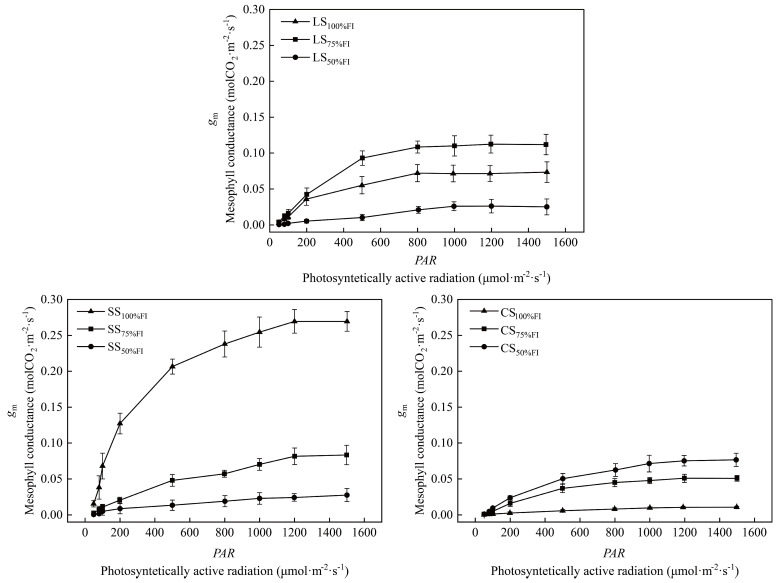
Differences in responses of mesophyll conductance (*g*_m_) of pear leaves to photosynthetically active radiation (*PAR*) among 9 treatments.

With the increase in photosynthetically active radiation (*PAR*), mesophyll conductance (*g*_m_) in all treatments increased initially, exhibiting a linear response, followed by a decreasing response. Then, the *g*_m_ reached its peak at the saturated *PAR* intensity (1500 μmol·m^−2^·s^−1^) (Figure 5). Leaf *g*_m_ in the sandy soil texture, combined with an irrigation level of 100% FI, was the most sensitive to the increase in *PAR*. The maximum *g*_m_ at the saturated *PAR* (*g*_m-max_) reached 0.271 molCO_2_·m^−2^·s^−1^, which was 2.2-fold and 8.7-fold higher than that of SS_75%FI_ and SS_50%FI_, respectively (*p* < 0.01). This indicated that sufficient irrigation in sandy soil can enhance the ability of leaves to utilize light energy, which is very important for compensating for the lack of soil water retention and maintaining photosynthetic efficiency. For the LS texture, the most sensitive *g*_m_ to *PAR* responded to the 75% FI irrigation level, and the *g*_m-max_ (0.115 molCO_2_·m^−2^·s^−1^) of LS_75%FI_ was 51.3% and 3.1-fold significantly higher than that of LS_100%FI_ and LS_50%FI_, respectively. Leaf *g*_m_ in CS decreased with the increase of irrigation level, and *g*_m-max_ (0.077 molCO_2_·m^−2^·s^−1^) of CS_50%FI_ was 48.1% higher than that of CS_75%FI_ significantly, while *g*_m-max_ of CS_75%FI_ was 3.7-fold higher than that of CS_100%FI_ (*p* < 0.05). These differences in response patterns indicated that the *SWC* environment, determined by soil texture, profoundly affected the construction of the CO_2_ transport pathway and its plasticity in response to changes in the leaf light environment.

### 2.7. Effects of Quantifying Values of α·β on g_m_ and Photosynthetic Biochemical Parameters

The values of the calculated maximum *g*_m_ at the saturated *PAR* (*g*_m_′_-max_ and *g*_m-max_), respectively, using the empirical value (0.425) and the quantified value (data derived from Figure 3) of *α·β* under different *SWC* are shown in Table 2. Compared to *g*_m-max_, *g*_m_′_-max_ was significantly overestimated (30.3% in LS_75%FI_) in the LS texture. Additionally, *g*_m_′_-max_ was generally underestimated in the SS texture, ranging from 8.8% (SS_50%FI_) to 26.4% (*p* < 0.05, SS_100%FI_). Accordingly, the widely adopted *α·β*’s empirical value has previously exerted a significant influence on the values of leaf *g*_m_ under different *SWC*, and our data demonstrated that the estimated deviation ranged from an underestimation of 26.4% (SS_100%FI_) to an overestimation of 30.3% (LS_75%FI_).

The values of the maximum Rubisco-limited rate of carboxylation (*V*_cmax-*C*i_, *V*_cmax-*C*c′_, and *V*_cmax-*C*c_), maximum rate of electron transport (*J*_max-*C*i_, *J*_max-*C*c′_, and *J*_max-*C*c_), and rate of triose-phosphate utilization (*V*_tpu-*C*i_, *V*_tpu-*C*c′_, and *V*_tpu-*C*c_) curve-fitted, respectively, from *A*_n_-*C*_i_ curves (*g*_m_ was not taken into account), *A*_n_-*C*_c_′ curves (empirical *α·β* for calculating *g*_m_), and *A*_n_-*C*_c_ curves (quantified *α·β* for calculating *g*_m_) of nine treatments are listed in Table 3. The three photosynthetic biochemical parameters curve-fitted using different methods under different *SWC* conditions showed that those of LS_75%FI_, SS_100%FI_, and CS_50%FI_ were significantly higher than those of other irrigation levels within the same texture. Compared with *V*_cmax-*C*c_, *J*_max-*C*c_, and *V*_tpu-*C*c_, neglecting *g*_m_ led to deviations in the estimated parameters. It ranged respectively from underestimation of 11.7% (CS_50%FI_) to overestimation of 5.8% (LS_75%FI_) for *V*_cmax-*C*i_, from an underestimation of 7.6% (CS_50%FI_) to an overestimation of 3.2% (LS_75%FI_) for *J*_max-*C*i_ and a consistent overestimation ranging from 11.1% (SS_100%FI_) to 46.2% (LS_75%FI_) for *V*_tpu-*C*i_ under the condition of no water stress (treatments LS_75%FI_, SS_100%FI_ and CS_50%FI_). Furthermore, the deviations, under drought or excessive soil moisture conditions (the other six treatments), increased significantly and resulted in the underestimation of 21.3% (in CS)~the overestimation of 10.2% (in SS) for *V*_cmax-*C*i_, the underestimation of 20.8% (in CS)~the overestimation of 15.0% (in SS) for *J*_max-*C*i_ and the unified overestimation of 27.7% (in CS)~53.1% (in SS) for *V*_tpu-*C*i_, respectively.

Although *g*_m_ was considered, our data showed that using the existing parametric values (*α·β* = 0.425) caused *V*_cmax-*C*c′_, *J*_max-*C*c′_, and *V*_tpu-*C*c′_ to fall into an underestimation of 6.4% (LS_75%FI_) up to an overestimation of 0.9% (CS_50%FI_), an underestimation of 5.7% (LS_75%FI_) up to an overestimation of 3.0% (CS_50%FI_), and an underestimation of 4.7% (SS_100%FI_) up to an overestimation of 15.5% (LS_75%FI_) compared to *V*_cmax-*C*c_, *J*_max-*C*c_, and *V*_tpu-*C*c_, respectively, when out of water stress (i.e., LS_75%FI_, SS_100%FI_ and CS_50%FI_). When encountering water stress events (drought or excessive soil moisture), however, *V*_cmax-*C*c′_, *J*_max-*C*c′,_ and *V*_tpu-*C*c′_ were underestimated by 7.7% (in LS)~22.2% (in CS), 2.2% (in SS)~28.8% (in CS), and 2.9% (in SS)~12.5% (in CS), respectively.

## 3. Discussion

### 3.1. Effects of Different Irrigation Levels on Soil Water Content Under Different Soil Textures

The physical properties of soil, such as texture and porosity, depend on the composition of different particle types, which in turn affect soil water retention, conductivity, and thus the availability of soil water [23]. The clay particles and silt particles in the soil had a larger specific surface area than the sand particles. Therefore, a higher clay content increased the retention capacity and availability of soil water, while a higher sand content weakened it [23,32]. Accordingly, soil water retention was significantly positively correlated with the content of clay and silt, while it was negatively correlated with sand content, thereby demonstrating a sequence (from high to low) of clay soil, loam soil, and sandy soil according to water retention capacity [33]. The results of Yang et al. [34] also supported the notion that soil texture is a significant factor in determining changes in soil moisture, with clay content exhibiting a positive correlation and sand content exhibiting a negative correlation with soil water content (*SWC*). Three types of soil texture (CS, SS and LS) were used in the present study, and the percentage of clay particles and silt particles in CS (75.29%) was 3.0-fold and 1.0-fold higher than that in SS (24.76%) and LS (74.73%), respectively. Thus, the water retention capacity of CS was significantly higher than that of SS. Therefore, the averaged *SWC* in CS during the experimental period was 85.4%, 92.4%, and 1.8-fold higher than that in SS under the irrigation levels of 100%FI, 75%FI, and 50%FI, respectively (Figure 1). Additionally, the kinetics of water conduction were also measured in this study. The data demonstrated that the diffusive and migratory characteristics of soil water in CS, SS, and LS were significantly different (Figure S1). For loam soil, *SWC* was lower in the shallow layer (0–20 cm) and higher in the deep layer (60–70 cm) before irrigation. *SWC* in the 0–20 cm soil layer increased significantly, and that in the 60–70 cm soil layer increased slightly 2 h after irrigation. *SWC* in the 30–40 cm soil layer began to increase at 4 h after irrigation; *SWC* in the 50–70 cm soil layer increased, while *SWC* in the 0–20 cm soil layer decreased to the level close to that at 24 h after irrigation. For sandy soil, lower *SWC* was in the shallow layer (0–20 cm) before irrigation. *SWC* in the 20–40 cm soil layer increased significantly 2 h after irrigation. Water infiltrated into the 40–70 cm soil layer, and the water points at the depth of 70 cm were obviously dense 4 h after irrigation. The water distribution of each soil layer tends to be uniform; the deep layer (70 cm) still maintains a high *SWC*, and the surface layer (0–20 cm) is concentrated again at 24 h after irrigation.

For clay soil, a lower *SWC* was found in the shallow layer (0–20 cm), a higher *SWC* in the deep layer (30–40 cm), and a small distribution of *SWC* in the 60–70 cm deep layer before irrigation. *SWC* in a 30–40 cm soil layer decreased gradually 2 h after irrigation. The water extends to the deep layer (50–70 cm) 4 h after irrigation. *SWC* in the 50–70 cm soil layer increased slightly 24 h after irrigation. Before irrigation, the spatial heterogeneity of the initial water distribution was dominated by texture differences (CS water holding capacity > LS > SS). At 2–4 h after irrigation, the water infiltration rate showed SS > LS > CS, consistent with soil permeability, as the macropore structure of SS accelerated water discharge, whereas the micropores of CS blocked water migration through capillary action (Figure S1). After 24 h of irrigation, the SS water distribution returned to the pre-irrigation state, and this irrigation level had the least effect on the vertical distribution. The water distribution of LS was the most uniform, and the higher the irrigation level, the higher the water content, resulting in LS_100%FI_ > LS_75%FI_ > LS_50%FI_. Due to the strong water-holding capacity of CS, water was retained in the surface layer (20–40 cm), and thus, deep migration was weak.

Soil texture’s regulation of *SWC* is a core intermediary pathway that affects plant photosynthetic parameters. The differences in water-holding characteristics across textures result in a heterogeneous water environment under the same irrigation strategy, which in turn leads to differential responses in leaf physiological and biochemical parameters (see Section 2.3 and Section 2.6 results).

### 3.2. Responses of Leaf Water Content (LWC) and Specific Leaf Area (SLA) to Soil Water Content Under Different Soil Textures

Specific leaf area (*SLA*) and leaf water content (*LWC*) are critical physiological indicators that reflect plants’ water status and resource allocation strategies [35,36,37]. Previous studies have reported that water stress leads to a significant decrease in both *SLA* and *LWC* [38,39,40]. Hang et al. [41] found that the *LWC* and *SLA* of five garden shrubs in South China were significantly reduced under drought conditions. Our study demonstrated that under low *SWC* conditions (50%FI), *SLA* decreased by 5.35% and 6.24%, and *LWC* decreased by 15.69% and 11.17%, respectively, compared to the higher *SWC* conditions of the 75%FI and 100%FI treatments (Figure 2). In line with previous findings, clay soil exhibits strong water retention but poor air permeability. When low *SWC* conditions occur, plants adapt to the imbalance between water supply and demand by reducing *SLA* and *LWC* (reducing water consumption). This strategy effectively minimizes water loss through transpiration and enhances drought resistance [42,43].

In a study examining the functional traits and environmental adaptation characteristics of *Ammopiptanthus mongolicus*, Li et al. [44] highlighted that specific *SLA* was closely linked to soil texture and moisture conditions. Regression analysis indicated a positive correlation between *SLA* and sand particle content in soil, suggesting that *SLA* was more likely to reach elevated levels in SS. The present study further revealed that *LWC* peaked at 75%FI, while *SLA* reached its maximum under the high *SWC* conditions of 100%FI in SS. This finding suggests that the low water retention capacity of sandy soil prompts plants to optimize leaf water storage under moderate moisture conditions [45]. Conversely, photosynthetic tissue, characterized by high *SLA*, is preferentially developed under high moisture conditions to mitigate nutrient limitations [46]. In LS, *SLA* exhibited an opposite trend to the typical drought response model (i.e., the more abundant water, the higher *SLA*) [47]. With increasing irrigation levels, *SLA* decreased continuously, and *LWC* at 75%FI was slightly higher than at 100%FI. This may be related to the water-holding and breathing balance characteristics of LS, and plants adopt thick-leaf defense strategies when water is sufficient. These findings demonstrated that soil texture affected the water status and resource allocation strategy of pear leaves by altering water availability, and plants adapted to different *SWC* environments resulting from soil-irrigation combinations by adjusting *LWC* and *SLA*, thereby providing a theoretical basis for understanding the water adaptation mechanism of fruit trees under different soil water conditions.

### 3.3. Responses of Parameter (α·β) and Mesophyll Conductance (g_m_) to Soil Water Content Under Different Soil Textures

The light absorption coefficient of plant leaves (*α*) and the light energy partitioning ratio between PSI and PSII (*β*) are crucial parameters for calculating mesophyll conductance (*g*_m_). *α* reflects the efficiency of the leaf capturing incident light, and *β* reflects the equilibrium state of electron transfer between the two photosynthesis systems. Nevertheless, existing reports used the empirical values of *α* (0.85) and *β* (0.5), and the product (*α*·*β*) was treated as a constant (0.425) [6]. Tian et al. [15] used a Miniature Leaf Spectrometer (CI-710) to measure nine mangrove plants and observed that the *α* values ranged from 0.88 to 0.92. However, the *β* value of 0.5 was still employed, so the *α*·*β* values varied from 0.44 to 0.46 in their study. However, the present study showed that not only were the *α*·*β* values significantly influenced by soil moisture conditions (soil texture and soil water content), but also that the range of *α*·*β* values was much wider than in existing published values. The *α*·*β* values in LS, SS, and CS were in the range of 0.297~0.660, 0.169~0.366, and 0.280~0.462, respectively (Figure 3), and the *α*·*β* values reached the peak under LS_75%FI_, SS_100%FI,_ and CS_50%FI_ treatments, which reflected the optimal state of light energy capture and distribution under non-stress conditions. Under low *SWC* conditions (such as SS_50%FI_ and LS_50%FI_), the *α*·*β* values decreased significantly (0.169 and 0.297, respectively). This may be because the low *SWC* in SS and LS caused leaf dehydration, which in turn damaged the thylakoid membrane to reduce light absorption efficiency (*α*) and disrupted the energy distribution balance between PSI and PSII (*β*). For clay, excessive irrigation (CS_100%FI_) would lead to poor soil ventilation, inhibition of root respiration and nutrient absorption, and even damage to chloroplast function, thereby reducing the *α*·*β* value (0.280). The existing literature reported that the *α* value varied slightly [15,48], indicating significant heterogeneity in *β* across species and ecological environments. Yin et al. [49] pointed out that accurately estimating *α*·*β* is essential for applying C_3_ plants (*Triticum aestivum*) photosynthesis models, and that the widely used empirical value of 0.425 is not a constant; instead, it varies with leaf physiological status, particularly leaf nitrogen content. Martins et al. [16] further showed that, even within a single species, different calibration approaches, such as those based on *A*_n_/*C*_i_ or *A*_n_/*PPFD* curves, can produce markedly different *α*·*β* values (0.36–0.64). They attributed these discrepancies primarily to differences in calibration data types rather than to species identity or specific leaf area. In our study, *α*·*β* values ranged from 0.169 to 0.660 across soil textures and irrigation levels, exceeding the variability previously reported in the literature. This indicates that *α*·*β* is influenced not only by species characteristics and calibration methodology but is also highly sensitive to the effective water availability in the rhizosphere, which is jointly determined by soil texture and irrigation regime. Our study further showed that quantifying the values of *α*·*β* was critical for the accurate calculation of *g*_m_. The previously used empirical value (0.425) caused *g*_m_ to fall into a deviation from an underestimation of 26.4% (SS_100%FI_) to an overestimation of 30.3% (LS_75%FI_) (Table 3).

Mesophyll conductance (*g*_m_) is the rate-limiting step of CO_2_ diffusion in leaves, which is different from *g*_s,_ which regulates the entry of CO_2_ into leaves. *g*_m_ controls the diffusion process of CO_2_ from the stomatal cavity to the chloroplast carboxylation site (*C*_c_) [6]. Its biological significance is to determine the actual CO_2_ concentration available to Rubisco (the photosynthetic core enzyme), which directly affects carbon assimilation efficiency. Some authors argue that the leaf *g*_m_ of plants grown under drought or salt stress is significantly lower than that of plants without unfavorable conditions [17,29,30]. Li et al. [31] reported that the mesophyll diffusion conductance of *Cotinus coggygria* seedlings was 25.3% higher under moderate stress (55~65% of Field Capacity) and 103.0% higher under severe stress (35~45% of FC), compared to the control (75~80% of FC). Their results also demonstrated that the effects of drought stress on leaf *g*_m_ of the seedlings were aggravated with stress duration, and *g*_m_ in the later stage of stress was 81.0% lower than that in the middle stage. Li et al. [50] designed five gradients of soil moisture (maintained at 40%, 50%, 60%, 70% and 80% of FC, respectively) in a potting experiment and observed that the stomatal conductance, mesophyll conductance, and the total conductance for CO_2_ transport of cucumber leaves decreased linearly with the reduction of soil water content. As the aggravation of soil water stress progressed, they concluded that the contribution of stomatal and mesophyll resistance to photosynthetic suppression increased progressively. The study by Flexas et al. [17] on grape plants showed that severe drought stress could reduce leaf *g*_m_ from 0.22 molCO_2_·m^−2^·s^−1^ to 0.02 molCO_2_·m^−2^·s^−1^ (i.e., a decrease of more than 90%). Our experimental results showed that the *g*_m-max_ values of leaves in sandy soil, loam soil, and clay soil without water stress (SS_100%FI_, LS_75%FI_, and CS_50%FI_) were 5.5-fold, 2.0-fold, and 3.4-fold higher (on average) than those of leaves in SS, LS, and CS under stress conditions, respectively. Therefore, it provided powerful evidence that mesophyll conductance was significantly different across soil textures and soil moisture conditions. In addition, the significant interaction between soil texture and irrigation on *g*_m_ (Table 1) was due to the combined regulation of leaf water status and cell structure. SS needs sufficient irrigation (100%FI) to maintain cell turgor, LS benefits from moderate irrigation (75%FI) to balance cell structure and gas exchange, and CS tolerates low irrigation (50%FI), which can help avoid anaerobic stress and excessive diffusion resistance.

Estimation deviation caused by the use of empirical *α*·*β* values will directly lead to misjudgments of plant water status and will thus affect the evaluation of irrigation strategies. For example, overestimating *g*_m_ under 75%FI in LS may mask its actual water diffusion limitation and be misleading in suggesting that additional irrigation is not needed; underestimating *g*_m_ under 100%FI in SS will weaken the correct evaluation of the importance of adequate irrigation for soil moisture conservation. Therefore, the quantitative *α*·*β* value serves as a basis for accurately evaluating leaf photosynthetic capacity and avoiding misattribution of key photosynthetic parameters.

In summary, this study shows that the core biological significance of the *g*_m_ difference across treatments is that it directly determines the supply of CO_2_ to Rubisco carboxylation sites, and this difference is primarily driven by *SWC* conditions. The decrease in *g*_m_ indicates that the diffusion resistance of CO_2_ from intercellular pores to chloroplasts increases, leading to a lower CO_2_ concentration (*C*_c_) at carboxylation sites. This not only limits Rubisco’s carboxylation efficiency but may also exacerbate photorespiration, thereby fundamentally restricting the photosynthetic potential of leaves. The highest *g*_m_ values observed in SS_100%FI_, LS_75%FI_, and CS_50%FI_ indicate that in these specific soil–water combinations, the CO_2_ transport path inside the pear leaves is the most unimpeded, and the mesophyll diffusion limitation of carbon assimilation is the smallest, thereby laying a physiological foundation for its maximum photosynthetic capacity. Our results strongly suggest that *g*_m_ is a sensitive and key physiological hub linking *SWC* with leaf photosynthetic and biochemical functions.

### 3.4. Responses of the Relationship Between g_m_ and PAR to Soil Water Content Under Different Soil Textures

An impressive body of literature has documented the response of *g*_m_ to light intensity under well-watered conditions [13,51]. Meng et al. [8] used an extended Farquhar model to explore the photosynthetic characteristics of nine tree species in northern subtropical regions and observed that leaf *g*_m_ of the trees decreased by 60.14% on average when *PAR* decreased from 1200 μmol·m^−2^·s^−1^ to 200 μmol·m^−2^·s^−1^. They claimed that the limited photosynthetic capacity of plants under low light intensity was due to the decreased *g*_m_ and *V*_tpu_. Hassiotou et al. [52] also reported that the leaf *g*_m_ of sclerophyllous plants under a light intensity of 1500 μmol·m^−2^·s^−1^ was approximately 22% higher than that under 500 μmol·m^−2^·s^−1^. Our data showed that the responses of leaf *g*_m_ to *PAR* under different *SWC* followed a rapid increase in weak light (*PAR* < 200 μmol·m^−2^·s^−1^), and then increased slowly in the *PAR* range of 200~800 μmol·m^−2^·s^−1^, finally stabilizing under higher light intensity (800 μmol·m^−2^·s^−1^ < *PAR* < 1500 μmol·m^−2^·s^−1^). Among the nine combinations of different soil textures and irrigation levels, the present study further demonstrated that leaf *g*_m_ of SS_100%FI_ was the most sensitive to the increase in *PAR*, and the *g*_m-max_ reached 0.271 molCO_2_·m^−2^·s^−1^ at saturated *PAR* (1500 μmol·m^−2^·s^−1^), while the lowest level of *g*_m-max_ decreased to 0.011 molCO_2_·m^−2^·s^−1^ under soil water stress (CS_100%FI_) (Figure 3). Accordingly, it can be safely concluded that the intensity of *g*_m_ responded to *PAR* differed significantly under both different soil textures and soil moisture conditions. Accordingly, it can be safely concluded that the intensity of *g*_m_ responded to *PAR* differed significantly under both different soil textures and soil moisture conditions.

### 3.5. Response of Photosynthetic Biochemical Parameters to Soil Water Content Under Different Soil Textures

A considerable majority of existing reports on the response of leaf photosynthetic rate to CO_2_ concentration failed to account for mesophyll cell resistance (i.e., assuming *C*_i_ = *C*_c_) [8,10], leading to deviations in estimates of leaf photosynthetic capacity. A classic example is Sun et al.’s [53] study, which found that by measuring and comparing nearly 130 C_3_ plants, the sensitivity of the three photosynthetic biochemical parameters to the changing *g*_m_ was *V*_cmax_ > *J*_max_ > *V*_tpu_, while the degree of underestimation of them could reach 75%, 60%, and 40% without accounting for *g*_m_. Wang et al. [54] found that the estimated leaf *A*_n_ of alfalfa plants was higher under higher salt stress (80 mmol·L^−1^·NaCl) than in lower concentrations (40 mmol·L^−1^) of NaCl if *g*_m_ was ignored. Meanwhile, researchers have also observed that accounting for *g*_m_ improves the estimation accuracy of leaf photosynthetic rate, with an increment of 0.81 to 0.93 in the coefficient of determination and a reduction in the mean absolute error from 27.5% to 24.3%.

The three key photosynthetic biochemical parameters (*V*_cmax_, *J*_max_ and *V*_tpu_) reflected the maximum rate of Rubisco carboxylation, the maximum rate of electron transport, and the triose-phosphate utilization rate, respectively. This study showed that the values of *V*_cmax_, *J*_max,_ and *V*_tpu_ in the LS_75%FI_, SS_100%FI,_ and CS_50%FI_ treatments were the highest (Table 3), indicating the optimal carbon assimilation ability. The present study demonstrated that neglecting *g*_m_ led to estimation deviations of −11.7% to 5.8% (negative and positive values represent underestimation and overestimation, respectively, as below), −7.6% to 3.2% and 11.1% to 46.2% for *V*_cmax_, *J*_max_, and *V*_tpu_, respectively, in the absence of water stress. The estimation deviation further increased to −21.3~10.2%, −20.8~15.0%, and 27.7~53.1% respectively, under drought or excessive soil-moisture conditions. Although *g*_m_ was considered, it was observed that using the existing empirical value resulted in estimation deviations of −6.4% to 0.9% (*V*_cmax_), −5.7% to 3.0% (*J*_max_), and −4.7% to 15.5% (*V*_tpu_) without water stress. In comparison, they were generally underestimated, resulting in unacceptable deviations of −22.2%, −28.8%, and −12.5% during water-stress events. Accordingly, quantifying key parameters under different edaphic conditions (e.g., varying soil water content, or *SWC*) is crucial for the accurate estimation of mesophyll conductance and photosynthetic biochemical parameters, and for the reliable evaluation of plant photosynthesis. It also provides an important theoretical basis for formulating accurate orchard irrigation strategies for different soil textures to achieve water savings and increased efficiency.

### 3.6. Physiological Mechanism of Soil Moisture Affecting Mesophyll Conductance and Photosynthetic Parameters

The differences in *g*_m_ and photosynthetic biochemical parameters observed under different soil textures and irrigation levels can be attributed to several physiological and structural mechanisms. First, *SWC* directly affects leaf turgor and cell wall elasticity, and then influences the porosity of the mesophyll air gap, which is the key determinant of CO_2_ diffusion resistance [5]. Under low *SWC* conditions, mesophyll cells usually undergo structural changes. Second, the differential response across soil textures highlights the role of soil hydraulic characteristics in regulating the resulting *SWC* and, consequently, plant water status. The low water-holding capacity of sand results in rapid decreases in *SWC*, leading to early pore closure and reduced *g*_m_ to conserve water. In contrast, clay has a longer water retention time, but this may lead to hypoxia under high irrigation, damaging mitochondrial ATP supply for respiration and photosynthetic processes. Loams have balanced water retention and air permeability, as observed in this study, allowing *g*_m_ to reach its best state under moderate irrigation (75%FI).

In addition, the product of light energy capture and utilization efficiency, *α·β*, varied widely across different *SWCs*, indicating that plants adjust their photosynthetic apparatus in response to soil-water availability. This plasticity helps optimize light energy utilization under stress, while underscoring the risk of using a fixed empirical *α·β* value in the model. These adaptation mechanisms not only explain differences in optimal irrigation levels across soil textures (LS_75%FI_, SS_100%FI_, CS_50%FI_) but also underscore the need to develop soil-specific water management strategies in orchard systems.

This study also has some limitations. First, the experiment was carried out under pot conditions. Although soil moisture was strictly controlled, it differed from the complex, changeable field environment. Second, the experimental materials used in this study were 5-year-old pear trees, and extrapolating the results to other tree ages or varieties requires careful verification. In addition, the experiment was conducted only during the growing season (May–June) and did not assess responses to water stress throughout the annual cycle, especially during key water-demanding stages such as fruit expansion. Future research can further examine the effects of soil texture, irrigation amount, and fertilization at different growth stages, and explore the interactions between physiological indicators, such as *g*_m_ and final fruit yield and quality, thereby constructing a more comprehensive water and fertilizer management model for pear orchards.

Ecologically, the differential responses of *g*_m_ and photosynthetic parameters to soil texture and irrigation level reflect the adaptation strategy of pear trees to a heterogeneous soil moisture environment. Pear trees are widely distributed across different soil types (plains, mountains, and river beaches) in China, and they can adjust *g*_m_, *α*·*β*, and biochemical parameters in response to soil water availability. For example, in SS orchards (such as floodplain areas), it is recommended to use sufficient irrigation (100%FI) to maintain high *g*_m_ and photosynthetic efficiency, and to make up for the lack of soil-water retention. For LS orchards (the most common soil type in the main pear-producing areas), moderate irrigation (75%FI) is the optimal choice, as it can balance CO_2_ diffusion, light energy utilization, and biochemical capacity, while achieving the dual goals of high yield and water savings. For CS orchards (e.g., poorly drained mountains), low irrigation (50%FI) should be used to avoid soil water accumulation and anaerobic stress, meanwhile improving soil structure to increase permeability and *g*_m_. In addition, the plasticity of photosynthetic regulation across different soil-irrigation combinations enables pear trees to cope with the variability of soil moisture conditions across ecological regions and also provides guidance for efficient water-saving irrigation, extensive cultivation, and high yield in pear orchards.

## 4. Materials and Methods

### 4.1. Experimental Site and Meteorological Conditions

**Figure 6:**
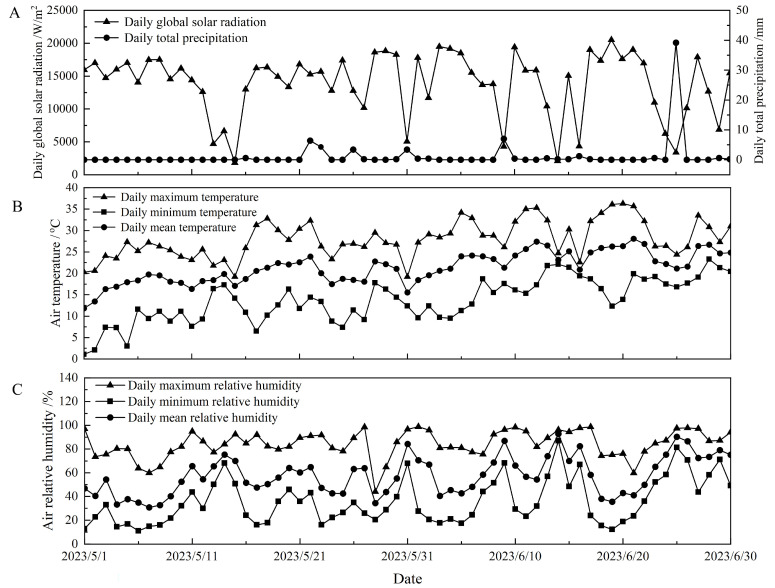
Daily global solar irradiation, total precipitation, and maximum, minimum, and mean value of air temperature and relative humidity at the experimental site from 1 May to 30 June 2023. (**A**) The cumulative total solar radiation and total precipitation were; (**B**) Daily maximum, minimum temperature, average temperature; (**C**) The average relative humidity.

The experiments were conducted at the Pear Research Site of the Institute of Forestry & Pomology, Beijing Academy of Agriculture & Forestry Sciences, Beijing, China (40.13° N, 116.65° E) from mid-May to mid-June in 2023. The site was 40 m above sea level and is characterized by a typical continental monsoon climate with annual mean precipitation of 550 mm, mean temperature of 10 °C, and total sunshine hours of 2792.3 h. Accumulated global solar radiation and total precipitation was 847,435 W/m^2^ and 69.6 mm respectively (Figure 6A), and averaged value of daily maximum, minimum and mean air temperature as well as mean relative humidity was 36.3 °C, 1.1 °C, 21.4 °C (Figure 6B), and 57.6% (Figure 6C), respectively, at the experimental site during the experiments (from 1 May to 30 June 2023) (Figure 6).

### 4.2. Experimental Design and Materials

**Figure 7:**
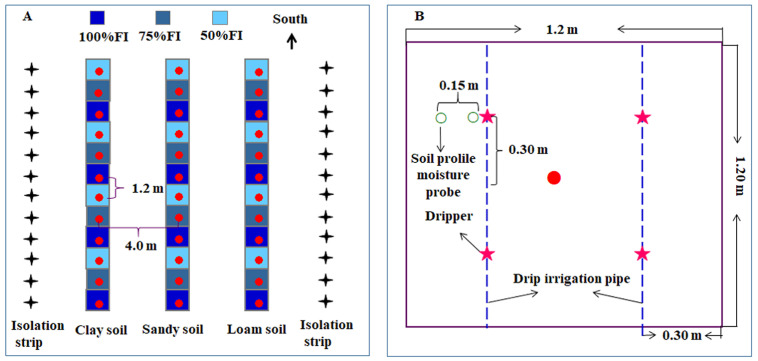
Field layout showing all experimental treatments (**A**) and fixed positions of drip irrigation pipes, drippers, and soil profile moisture probes in each potted container (**B**).

In the spring of 2018, 36 potted containers (cube-shaped with a side length of 1.2 m) were placed 0.2 m above the ground and arranged into three rows (12 in each row) along the north–south direction with a row spacing of 4.00 m (Figure 7A). Three types of soil texture, i.e., clay soil (CS), sandy soil (SS), and loam soil (LS), were filled (1.584 m^3^/pot) in three rows of containers (one row corresponded to one type). The percentages of clay, silt, and sand in CS, SS, and LS, along with their field capacities (*v*/*v*), are listed in Table 4. Five-year-old ‘Huangguan’ pear trees with uniform height, crown width, and growth vigor were chosen as experimental materials, and one of them was planted at the center of each container; thus, the experimental trees had a density of 1.20 m (plant) × 4.00 m (row). Two rows of drip irrigation pipes were arranged on both sides of the tree line at a distance of 0.30 m from the tree line, and one electromagnetic valve was installed at the north end of each drip irrigation pipe. In each potted container, four drippers were installed on the drip irrigation pipe, with each dripper located 0.30 m from the tree intersection, perpendicular to the drip irrigation pipe. To measure soil water content, four 1.0 m long soil profile moisture probes were set 0.9 m below the ground and located, respectively, two under the dripper and two 0.15 m away from the dripper, in a vertical direction relative to the drip irrigation pipe, in each potted container. The positions of drip irrigation pipes, drippers, and soil profile moisture probes in each potted container are shown in Figure 7B.

### 4.3. Experimental Treatment and Irrigation

Three irrigation levels (100% (CK), 75% and 50% of full irrigation) for each of the three soil textures (CS, SS, and LS) were conducted in late March 2023. Therefore, a total of nine treatments were set up in this experiment, which were denoted as CS_100%FI_(CK), CS_75%FI_, CS_50%FI_, SS_100%FI_(CK), SS_75%FI_, SS_50%FI_, LS_100%FI_(CK), LS_75%FI_, and LS_50%FI_, with four replicates (i.e., four potted containers) for each treatment. All treatments were irrigated when the 100% field irrigation (FI) reached the lower limit of irrigation (soil water content reached 85% of field capacity), and irrigation ceased when the FI reached the upper limit of irrigation (SWC reached 100% field capacity). The irrigation duration can be calculated by the irrigation level (converted based on the parameters such as FC and soil volume) and the rate of water flow from the dripper. Accordingly, different irrigation levels were applied based on varying water flow rates (100%FI, 75%FI, and 50%FI were 4 L/h, 3 L/h, and 2 L/h, respectively), while the same irrigation duration was maintained. In every spring season before germination (usually in late March), sufficient and the same amount of nitrogen-phosphorus-potassium compound fertilizers and micro fertilizers were applied to each potted container to ensure the normal growth of the tree (i.e., the nutrient was not a limiting factor for tree growth). Other management measures were the same as those used for commercial production.

### 4.4. Measurements

#### 4.4.1. Soil Water Content (SWC)

During the experimental period (from May to June 2023), the water content of nine treatments in the 0~0.4 m soil layer (i.e., the root layer of pear trees, divided into four layers of 0~0.1 m, 0.1~0.2 m, 0.2~0.3 m, and 0.3~0.4 m, respectively, the averaged value for the analysis) was measured every 2 days using an HD2 portable soil moisture measuring instrument (IMKO Co., Ltd., Ettlingen, Germany). It was kept consistent in all cultivation pools. Each treatment (texture and irrigation combination) was repeated three times, and each cultivation pool was repeated once. Before the experiment, the instrument was calibrated using the drying method: for the three soils—clay, sand, and loam—the soil samples with different water contents in the 0~90 cm soil layer were collected using the soil drilling method, and the soil volumetric water content of the corresponding soil layer was measured using HD2. By comparing the measured HD2 values with the actual water content determined by drying at 105 °C for 24 h, calibration curves for the three soil textures were established, and soil-type corrections were applied accordingly.

#### 4.4.2. Leaf Water Content (LWC) and Specific Leaf Area (SLA)

From mid-May to mid-June 2023, three trees were selected for each treatment, and three mature, healthy, and intact leaves were randomly chosen on 1-year-old shoots from each tree to measure their fresh weight. A leaf area meter (YMJ-C type, ZHE JIANG TOP INSTRUMENT Co., Ltd., Wenzhou, China) was used to scan and record the leaf area piece by piece. The leaves were then placed in an envelope and subsequently dried in an oven at 105 °C for 30 min. After this initial drying, the leaves were further dried to a constant weight at 80 °C, and the dry weight was measured to determine the water content of the leaf samples.

Leaf water content (*LWC*) = (Leaf fresh weight − Leaf dry weight)/Leaf fresh weight × 100%

Specific leaf area (*SLA*) = Leaf area/Leaf dry weight

#### 4.4.3. Mesophyll Conductance (*g*_m_)

In this study, gas exchange combined with chlorophyll fluorescence was used to determine mesophyll conductance (*g*_m_). Using the Farquhar model [55] and the quantum yield of photosystem II (PSII) based on chlorophyll fluorescence measurement [56], *g*_m_ is expressed as:

(1) $$g_{m}=\frac{A_{n}}{C_{i}-\frac{\Gamma^{*}\left[J_{f}+8(A_{n}+R_{d})\right]}{J_{f}-4(A_{n}+R_{d})}},$$

where *g*_m_ is mesophyll conductance (molCO_2_·m^−2^·s^−1^), *A*_n_ is net photosynthetic rate (μmolCO_2_·m^−2^·s^−1^), *C*_i_ is intercellular CO_2_ concentration (µmol·mol^−1^), Γ* is CO_2_ compensation point of dark respiration under light (μmol·mol^−1^), *J*_f_ is rate of electron transfer (μmol·m^−2^·s^−1^), and *R*_d_ is dark respiration rate under light (μmol·m^−2^·s^−1^). Equation (2) [57] and Equation (3) [56] can calculate Γ* and *J*_f_:

Γ* = *C*_i_* + *R*_d_/*g*_m,_ (2)

where *C*_i_* is apparent CO_2_ compensation point (μmol·mol^−1^).

*J*_f_ = *α·β*·*PAR*·*Φ*_PSII,_ (3)

where *α* is the light absorption coefficient of leaves, *β* is the light energy partitioning ratio between PSI and PSII, *Φ*_PSII_ the is photochemical efficiency of PSII, and *PAR* is photosynthetically active radiation (μmol·m^−2^·s^−1^).

Combing Equations (1)–(3), *g*_m_ can be expressed as (after converting Γ* to *C*_i_*):

(4) $$g_{m}=\frac{A_{n}\lfloor J_{f}-4(A_{n}+R_{d})\rfloor +R_{d}\lfloor J_{f}+8(A_{n}+R_{d})\rfloor }{C_{i}\left[J_{f}-4(A_{n}+R_{d})\right]-C_{i}^{*}\left[J_{f}+8(A_{n}+R_{d})\right]}$$

To quantify the effect of soil water content on mesophyll conductance, the parameters *Φ*_PSII_, *α·β*, *C*_i_*, and *R*_d_ were measured under different soil water conditions as follows.

#### 4.4.4. Response Curves of Φ_PSII_ to PAR

Measurements were conducted from 08:30 am to 11:30 pm on clear days from mid-May to mid-June 2023. For each tree in the potted container, one newly fully expanded, exposed, non-senescing, and healthy leaf (the 6th to 9th leaf counting from the tip) at growing shoots on the external of the canopy on the east side was randomly selected for measurements. Each leaf was measured only once (i.e., each treatment [texture and irrigation combination] was repeated three times; a total of 27 leaves were measured). The same as was performed below. The response curves of *Φ*_PSII_ to *PAR* were measured using an open gas exchange system (LI-6400XT, LI-COR Biosciences Inc., Lincoln, NE, USA) equipped with an integrated fluorescence chamber head (LI-6400-40). During measurements, *PAR* was set in a decreasing series (1600, 1400, 1200, 1000, 800, 600, 400, 200, 100, 80, 60, 40, 20, and 0 μmol·m^−2^·s^−1^). At the same time, CO_2_ concentration in the sample chamber (*C*_s_), temperature, and air humidity within the leaf chamber were adjusted to 400 μmol·mol^−1^ (controlled by CO_2_ injection system), (25 ± 1) °C, and 60% ± 5%, respectively. The recorded *F*_m_′ (maximum fluorescence yield under saturated pulse-activated light) and *F*_s_ (steady-state fluorescence yield under pulsed-activated light) were used to calculate *Φ*_PSII_ (*Φ*_PSII_ = (*F*_m_′ − *F*_s_)/*F*_m_′).

#### 4.4.5. Product of Light Absorption Coefficient and Light Energy Partitioning Ratio (α·β)

For the product of light absorption coefficient and light energy distribution ratio (*α*·*β*), the Valentini method [58] was used to determine the light response curve of the leaves under normal (21% O_2_) and low (2% O_2_) oxygen conditions. Under normal oxygen conditions (21% O_2_), the relative humidity in the leaf chamber was (400 ± 20) μmol·mol^−1^, and the flow rate was set to 300 μmol·mol^−1^. For the measurement under low oxygen conditions (2% O_2_), a mixed gas containing 2% O_2_ and 400 ± 20 μmol·mol^−1^ CO_2_ was supplied using a pure N_2_ steel bottle. The flow rate was set to 300 μmol·mol^−1^, and the outlet of the mixed gas of pure N_2_ steel bottle and CO_2_ was connected to the inlet of the air buffer bottle. The photochemical efficiency of photosystem II (*Φ*_PSII_) and the CO_2_ assimilation efficiency (*Φ*_CO2_) of the leaves were measured, respectively. By linear-fitting *Φ*_CO2_ versus *Φ*_PSII_, the slope of the fitted line was approximately equal to the value of *α*·*β* [49].

#### 4.4.6. Apparent CO_2_ Compensation Point (*C*_i_*) and Dark Respiration Rate Under Light (R_d_)

This study employed the widely used approach, modified by Sun et al. [7] and based on Laisk’s method [59], to measure *C*_i_* and *R*_d_. Under three low light intensities (*PAR* was set as 150, 100 and 50 μmol·m^−2^·s^−1^, respectively), the *A*_n_-*C*_i_ response curves of the measured leaves of each treatment were measured under low CO_2_ concentration (*C*_s_ was set as 150, 120, 90, 70 and 50 µmol·mol^−1^, respectively) using LI-6400XT with its standard 6-cm^2^ leaf chamber and red-blue LED light source (LI-6400-02B). During measurements, the temperature and air humidity within the leaf chamber were adjusted to the same conditions as mentioned above. By linear-fitting *A*_n_ versus *C*_i_, the three fitted lines intersected to form a triangular area. The intercepts of barycenter (i.e., the intersection of the three middle lines) of the triangle area at *X*-axis and *Y*-axis were approximately to the *C*_i_* value and the *R*_d_ value, respectively.

#### 4.4.7. A_n_-C_i_ and A_n_-C_c_ Response Curves and Determination of Photosynthetic Biochemical Parameter

For the measurements of *A*_n_-*C*_i_ response curves, *PAR* was set as 1500 μmol·m^−2^·s^−1^ (saturated *PAR* derived from light response curves), and *C*_s_ was sequentially set at 400, 300, 200, 100, 400, 600, 800, 1000, 1200, 1500, and 2000 μmol·mol^−1^. The temperature and air humidity within the leaf chamber were adjusted to the same conditions as mentioned above during measurements. According to the law of gas diffusion in leaves and the definition of *g*_m_, the CO_2_ concentration at the carboxylation site in the chloroplast (*C*_c_) can be calculated (*C*_c_ = *C*_i_-*A*_n_/*g*_m_). Thus, the *A*_n_-*C*_c_ response curves can be plotted. The *A*_n_-*C*_i_ and *A*_n_-*C*_c_ response curves were created to determine photosynthetic biochemical parameters, including the maximum Rubisco-limited rate of carboxylation (*V*_cmax_), the maximum rate of electron transport under light saturation (*J*_max_), and the rate of triose-phosphate utilization (*V*_tpu_), based on the FvCB model [60].

### 4.5. Data Processing and Statistical Analyses

Calculation, plotting, and linear regressions were performed using Origin 2023. Non-linear fitting was conducted using the GAUSS method in PROC NWM LIN of SAS 9.4 (SAS Institute Inc., Cary, NC, USA). Statistical analysis of treatment differences was performed using SPSS software (Windows version 22.0; SPSS, Chicago, IL, USA). A two-way ANOVA was used to analyze the effects of soil texture and irrigation amount on photosynthetic parameters. Before the analysis, the Shapiro–Wilk test was used to assess normality, and the Levene test was used to assess homogeneity of variance. All data met the assumptions of variance analysis. The post hoc test was performed using the least significant difference (LSD) method at the significance level of *p* < 0.05. Each combination of soil texture–irrigation levels contained four replicates (four trees were randomly selected, and one leaf was measured for each tree, *n* = 3); the total sample size was 27 trees. All measurements were performed on these independent individuals. To avoid false repetition, a repeated-measures ANOVA was used to analyze soil water content data collected at different time points, with time as the intra-group factor and treatment as the inter-group factor.

## 5. Conclusions

Mesophyll conductance (*g*_m_) and its key parameters, such as the product of light absorption coefficient and light energy partitioning ratio (*α·β*), apparent CO_2_ compensation point (*C*_i_*), and dark respiration rate under light (*R*_d_) of pear trees, showed significant divergences in responses to the changing soil water content at different textures. Using the existing empirical *α·β* value caused estimation deviations of −26.4% to 30.3% for *g*_m_, and underestimations of 22.2%, 28.8%, and 12.5% for *V*_cmax_, *J*_max_, and *V*_tpu_, respectively, under water stress conditions. The maximum leaf *g*_m_ could be obtained at 75%FI for loam soil, 100%FI for sandy soil, and 50%FI for clay soil, respectively. However, determining the optimal irrigation level across different soil textures to guide pear production will require the study and integration of the effects of various soil water contents on pear tree yield and fruit quality in the future.

## Figures and Tables

**Table 1:** Results (*F* and *p* value) of two-way ANOVA on the effects of soil textures and irrigation levels on *g*_m_ and related parameters. Significance at *p* < 0.05 is presented in bold.

Dependent Variable		Soil Textures	Irrigation Levels	Soil Textures × Irrigation Levels
*g*_m_	*F*	112.2	143.7	164.1
	*p*	**<0.001**	**<0.001**	**<0.001**
*α·β*	*F*	2.129	52.54	4.074
	*p*	0.148	**<0.001**	**0.0159**
*C*_i_*	*F*	36.44	63.48	26.78
	*p*	**<0.001**	**<0.001**	**<0.001**
*R*_d_	*F*	105.5	78.66	132.6
	*p*	**<0.001**	**<0.001**	**<0.001**
*V*_cmax_	*F*	11.23	4.057	29.34
	*p*	**0.018**	0.1403	**<0.001**
*J*_max_	*F*	18.58	5.876	37.75
	*p*	**0.0111**	0.0917	**<0.001**
*V*_tpu_	*F*	0.2072	1.026	25.62

Note: *g*_m_, mesophyll conductance; *α·β*, the product of light absorption coefficient and light energy partitioning ratio; *C*_i_*, apparent CO_2_ compensation point; *R*_d_, dark respiration rate under light; *V*_cmax_, maximum Rubisco-limited rate of carboxylation; *J*_max_, maximum rate of electron transport; *V*_tpu_, rate of triose-phosphate utilized.

**Table 2:** Differences in maximum mesophyll conductance under saturated *PAR* (1500 μmol·m^−2^·s^−1^), respectively, using the empirical *α·β* value (0.425) (*g*_m_′_-max_) and quantified *α·β* value (data from Figure 3) (*g*_m-max_) of different treatments.

Mesophyll Conductance (molCO_2_·m^−2^·s^−1^)	LS_100%FI_	LS_75%FI_	LS_50%FI_	SS_100%FI_	SS_75%FI_	SS_50%FI_	CS_100%FI_	CS_75%FI_	CS_50%FI_
*g*_m_′_-max_	0.086 ± 0.015 a	0.150 ± 0.020 a	0.027 ± 0.007 a	0.199 ± 0.005 a	0.075 ± 0.009 b	0.025 ± 0.011 a	0.011 ± 0.001 a	0.051 ± 0.005 a	0.080 ± 0.009 a
*g*_m-max_	0.076 ± 0.015 a	0.115 ± 0.013 b	0.028 ± 0.008 a	0.271 ± 0.015 b	0.084 ± 0.013 a	0.028 ± 0.009 a	0.011 ± 0.001 a	0.052 ± 0.005 a	0.077 ± 0.008 a

Note: Different letters in the same column indicate a significant difference at the 0.05 level between *g*_m_′_-max_ and *g*_m-max_ of each treatment.

**Table 3:** Maximum Rubisco-limited rate of carboxylation (*V*_cmax_), maximum rate of electron transport (*J*_max_), and rate of triose-phosphate utilization (*V*_tpu_), derived from *A*_n_-*C*_i_ curves, *A*_n_-*C*_c_′ curves (empirical *α·β*), and *A*_n_-*C*_c_ curves (quantified *α·β*) of different treatments (μmol·m^−2^·s^−1^), respectively.

Treatments	Derived from *A*_n_-*C*_i_ Curves	Derived from *A*_n_-*C*_c_′ Curves (Empirical *α·β*)	Derived from *A*_n_-*C*_c_ Curves (Quantified *α·β*)
	*V*_cmax-*C*i_	*V*_cmax-*C*c’_	*V*_cmax-*C*c_
LS_100%FI_	96.1 ± 8.5 b	88.2 ± 3.9 a	91.4 ± 9.2 ab
LS_75%FI_	110.6 ± 4.6 a	97.8 ± 8.0 a	104.6 ± 6.3 a
LS_50%FI_	88.9 ± 9.5 b	75.7 ± 6.4 b	85.9 ± 9.2 b
SS_100%FI_	84.7 ± 5.7 a	90.3 ± 3.7 a	90.1 ± 9.7 a
SS_75%FI_	80.2 ± 6.2 a	74.8 ± 7.8 b	75.9 ± 7.4 ab
SS_50%FI_	77.0 ± 5.7 a	52.3 ± 2.4 c	67.2 ± 7.5 b
CS_100%FI_	28.8 ± 2.7 b	25.3 ± 2.4 c	41.3 ± 4.7 b
CS_75%FI_	110.5 ± 11.2 a	118.9 ± 9.1 b	126.0 ± 11.6 a
CS_50%FI_	121.4 ± 5.4 a	138.8 ± 5.6 a	137.5 ± 14.3 a
**Treatments**	***J*_max-*C*i_**	***J*_max-*C*c′_**	***J*_max-*C*c_**
LS_100%FI_	95.5 ± 10.8 ab	73.4 ± 5.0 b	92.2 ± 8.3 a
LS_75%FI_	106.6 ± 7.0 a	97.3 ± 6.9 a	103.2 ± 10.0 a
LS_50%FI_	86.5 ± 8.2 b	60.6 ± 2.7 c	77.7 ± 4.4 b
SS_100%FI_	87.9 ± 7.3 a	90.9 ± 5.6 a	90.5 ± 5.7 a
SS_75%FI_	84.1 ± 3.9 a	68.0 ± 3.5 b	66.8 ± 7.1 b
SS_50%FI_	63.0 ± 7.0 b	56.8 ± 8.6 b	60.5 ± 5.3 b
CS_100%FI_	26.9 ± 3.4 b	22.2 ± 1.7 c	39.8 ± 4.6 b
CS_75%FI_	120.8 ± 6.6 a	115.2 ± 10.2 b	133.3 ± 9.4 a
CS_50%FI_	125.4 ± 7.7 a	139.7 ± 5.5 a	135.7 ± 12.8 a
**Treatments**	***V*_tpu-*C*i_**	***V*_tpu-*C*c′_**	***V*_tpu-*C*c_**
LS_100%FI_	13.8 ± 0.4 a	9.0 ± 0.5 b	9.6 ± 0.2 a
LS_75%FI_	14.3 ± 1.2 a	11.3 ± 1.5 a	9.7 ± 1.5 a
LS_50%FI_	7.7 ± 1.5 b	4.4 ± 1.3 c	4.7 ± 1.4 b
SS_100%FI_	12.8 ± 2.5 a	11.0 ± 2.2 a	11.6 ± 2.3 a
SS_75%FI_	10.2 ± 0.8 a	6.8 ± 1.3 b	6.7 ± 0.8 b
SS_50%FI_	6.7 ± 1.8 b	4.1 ± 0.9 c	4.4 ± 1.0 c
CS_100%FI_	5.3 ± 1.6 c	3.3 ± 1.1 c	4.1 ± 1.6 c
CS_75%FI_	9.5 ± 1.1 b	7.1 ± 1.4 b	7.5 ± 1.4 b
CS_50%FI_	15.1 ± 1.2 a	12.0 ± 0.8 a	11.8 ± 0.9 a

Note: Different letters in the same column indicate a significant difference at the 0.05 level among treatments of different irrigation levels under the same soil texture.

**Table 4:** Percentages of clay, silt, and sand in different types of soil textures used in this study.

Types of Soil Texture	Clay Particle (%)	Silt Particle (%)	Sand Particle (%)	Field Capacity (*v*/*v*, %)
Clay soil (CS)	18.58	56.71	24.73	28.1
Sandy soil (SS)	1.99	22.77	75.24	15.9
Loam soil (LS)	4.59	70.14	25.24	22.8

## Data Availability

The original contributions presented in this study are included in the article/supplementary material. Further inquiries can be directed to the corresponding author.

## Supplementary Materials

The following supporting information can be downloaded at: https://www.mdpi.com/article/10.3390/plants14243784/s1.

## References

[1] Flexas J., Ribas-Carbo M., Diaz-Espejo A., Galmés J., Medrano H. (2008). Mesophyll conductance to CO_2_: Current knowledge and prospects. Plant Cell Environ..

[2] Zhang X.Y., Huang Z.Y., Su X.H., Siu A., Song Y.P., Zhang D.Q., Fang Q. (2020). Machine learning models for net photosynthetic rate prediction using poplar leaf phenotype data. PLoS ONE.

[3] Hou B.P., Wang J.Q., Li F.Q., Shi J., Gao J., Shen D.Y., Li K.Y. (2025). Hyperspectral estimation modeling of photosynthetic rate in cotton canopy using chlorophyll data. Agric. Res. Arid. Areas.

[4] Hasper T.B., Dusenge M.E., Breuer F., Uwizeye F.K., Wallin G., Uddling J. (2017). Stomatal CO_2_ responsiveness and photosynthetic capacity of tropical woody species about taxonomy and functional traits. Oecologia.

[5] Flexas J., Barbour M.M., Brendel O., Cabrera H.M., Carriquí M., Díaz-Espejo A., Douthe C., Dreyer E., Ferrio J.P., Gago J. (2012). Mesophyll diffusion conductance to CO_2_: An unappreciated central player in photosynthesis. Plant Sci..

[6] Zhu K., Yuan F.H., Guan D.X., Wu J.B., Wang A.Z. (2019). Measuring and calculating methods of plant mesophyll conductance: A review. Chin. J. Appl. Ecol..

[7] Sun Y., Gu L., Dickinson R.E., Pallardy S.G., Baker J., Cao Y., DaMatta F.M., Dong X., Ellsworth D., van Goethem D. (2014). Asymmetrical effects of mesophyll conductance on fundamental photosynthetic parameters and their relationships estimated from leaf gas exchange measurements. Plant Cell Environ..

[8] Meng J.L., Zhou B.Z., Cao Y.H., Yang L.J. (2016). Limiting states of photosynthesis of common tree species in the north-subtropical forest based on the improved Farquhar model. J. Trop. Subtrop. Bot..

[9] Lin L., Li Z.Q., Yu L., Wang H.N., Niu Z.M. (2020). Photosynthetic responses to the interaction of light intensity and CO_2_ concentration and photoinhibition characteristics of two apple canopy shapes. Acta Hortic. Sin..

[10] Lin L., Niu Z.M., Jiang C.D., Yu L., Wang H.N., Qiao M.Y. (2022). Influences of open-central canopy on photosynthetic parameters and fruit quality of apples (*Malus domestica*) in the Loess Plateau of China. Hortic. Plant J..

[11] Ethier G., Livingston N. (2004). On the need to incorporate sensitivity to CO_2_ transfer conductance into the Farquhar-von Caemmerer-Berry leaf photosynthesis model. Plant Cell Environ..

[12] Xu C., Liu X.L., Li Q., Ling F.L., Wu Z.H., Zhang Z.A. (2018). Effect of salt stress on photosynthesis and chlorophyll fluorescence characteristics of rice leaves for nitrogen levels. Chin. Bull. Bot..

[13] Liu J., Hu X.T., Wang W.E., Ran H., Fang S.L., Yang X. (2019). Effects of light intensity and photoperiod on photosynthetic characteristics and chlorophyll fluorescence of hydroponic lettuce. Southwest China J. Agric. Sci..

[14] Laisk A., Loreto F. (1996). Determining photosynthetic parameters from leaf CO_2_ exchange and chlorophyll fluorescence (Ribulose-1,5-bisphosphate carboxylase/oxygenase specificity factor, dark respiration in the light, excitation distribution between photosystems, alternative electron transport rate, and mesophyll diffusion resistance. Plant Physiol..

[15] Tian S.Q., Zhu S.D., Zhu J.J., Shen Z.H., Cao K.F. (2016). Impact of leaf morphological and anatomical traits on mesophyll conductance and leaf hydraulic conductance in mangrove plants. Plant Sci. J..

[16] Martins S.C., Galmés J., Molins A., DaMatta F.M. (2013). Improving the estimation of mesophyll conductance to CO_2_: On the role of electron transport rate correction and respiration. J. Exp. Bot..

[17] Keenan T., Sabate S., Gracia C. (2010). Soil water and coupled photosynthesis-conductance models: Bridging the gap between conflicting reports on the relative roles of stomatal, mesophyll conductance, and biochemical limitations to photosynthesis. Agric. For. Meteorol..

[18] Wang R.R., Xia J.B., Yang J.H., Zhao Y.Y., Liu J.T., Sun J.K. (2013). Comparison of light response models of photosynthesis in leaves of *Periploca sepium* under drought stress in a sand habitat formed from seashells. Chin. J. Plant Ecol..

[19] Zuo Y.M., Chen Q.B., Deng Q.Q., Tang J., Luo H.W., Wu T.K., Yang Z.F. (2011). Effects of soil moisture, light, and air humidity on stomatal conductance of cassava (*Manihot esculenta* Crantz). Chin. J. Ecol..

[20] Cui X.Y., Song J.F., Zhang Y.H. (2004). Some photosynthetic characteristics of *Fraxinus Mandshurica* seedlings grown under different soil water potentials. Acta Phytoecol. Sin..

[21] Campos H., Trejo C., Peña-Valdivia C.B., García-Nava R., Conde-Martínez F.V., Cruz-Ortega M.R. (2014). Stomatal and nonstomatal limitations of bell pepper (*Capsicum annuum* L.) plants under water stress and rewatering: Delayed restoration of photosynthesis during recovery. Environ. Exp. Bot..

[22] Wosten J.H.M., Pachepsky Y.A., Rawls W.J. (2001). Pedotransfer functions: Bridging the gap between available basic soil data and missing soil hydraulic characteristics. J. Hydrol..

[23] Li H., Yan F.C., Jiao J.Y., Tang B.Z., Zhang Y.F. (2018). Soil water availability and holding capacity of different vegetation types in the hilly-gullied region of the Loess Plateau. Acta Ecol. Sin..

[24] Kool D., Tong B., Tian Z., Heitman J.L., Sauer T.J., Horton R. (2019). Soil water retention and hydraulic conductivity dynamics following tillage. Soil. Tillage Res..

[25] Han J.M., Lei Z.Y., Zhang Y.J., Yi X.P., Zhang W.F., Zhang Y.L. (2019). Drought-introduced variability of mesophyll conductance in Gossypium and its relationship with leaf anatomy. Physiol. Plant.

[26] Alonso-Forn D., Peguero-Pina J.J., Ferrio J.P., Mencuccini M., Mendoza-Herrer Ó., Sancho-Knapik D., Gil-Pelegrín E. (2021). Contrasting functional strategies following severe drought in two Mediterranean oaks with different leaf habits: Quercus faginea and Quercus ilex subsp. rotundifolia. Tree Physiol..

[27] Zou J., Hu W., Li Y.X., Zhu H.H., He J.Q., Wang Y.H., Meng Y.L., Chen B.L., Zhao W.Q., Wang S.S. (2022). Leaf anatomical alterations reduce cotton’s mesophyll conductance under dynamic drought stress conditions. Plant J..

[28] Wang L., Zhang T., Ding S.Y. (2009). Effect of Short-term Drought and Rewatering During the Blooming Stage on Soybean Photosynthesis and Yield. Chin. Bull. Bot..

[29] Perez-Martin A., Michelazzo C., Torres-Ruiz J.M., Flexas J., Fernández J.E., Sebastiani L., Diaz-Espejo A. (2014). Regulation of photosynthesis and stomatal and mesophyll conductance under water stress and recovery in olive trees: Correlation with gene expression of carbonic anhydrase and aquaporins. J. Exp. Bot..

[30] Chen T.W., Kahlen K., Stützel H. (2015). Disentangling the contributions of osmotic and ionic effects of salinity on stomatal, mesophyll, biochemical, and light limitations to photosynthesis. Plant Cell Environ..

[31] Li J.H., Qi X.H., Xu C.Y., Wang C., Liu H.X., Sun P. (2015). Short-term responses of leaf gas exchange characteristics to drought stress of *Cotinus coggygria* seedlings. Sci. Silvae Sin..

[32] Zheng C.D., Cheng Y., Zhang M.M. (2014). Effect of Soil Texture on Soil Physical Properties and Regulations. J. Arid Land Resour. Environ..

[33] Liu Z.P., Ma D.H., Hu W., Li X.L. (2018). Land use dependent variation of soil water infiltration characteristics and their scale-specific controls. Soil Tillage Res..

[34] Yang M., Zhao X.N., Gao X.D., Yang S.W. (2019). Deep soil moisture use of planted forests in the Loess Plateau. Res. Soil Water Conserv..

[35] Májeková M., Martínková J., Hájek T. (2019). Grassland plants show no relationship between leaf drought tolerance and soil moisture affinity, but rapidly adjust to changes in soil moisture. Funct. Ecol..

[36] Wei H.X., Dong Q.Z., Zhou Z.K., Zhang Z.G. (2022). Leaf traits of the main plant species under different habitat conditions in the Mu Us Sandy Land. J. Arid Land Resour. Environ..

[37] Zununjan Z., Turghan M.A., Sattar M., Kasim N., Emin B., Abliz A. (2024). Combining the fractional order derivative and machine learning for leaf water content estimation of spring wheat using hyperspectral indices. Plant Methods.

[38] Wellstein C., Poschlod P., Gohlke A., Chelli S., Campetella G., Rosbakh S., Canullo R., Kreyling J., Jentsch A., Beierkuhnlein C. (2017). Effects of extreme drought on specific leaf area of grassland species: A meta-analysis of experimental studies in temperate and sub-Mediterranean systems. Glob. Change Biol..

[39] Zhang Q.H., Xiang F.Y., Zeng X.G., Han Y.C., Guo C., Gu Y.C., Chen F.Y. (2018). Physiological Response of Different Strawberry Cultivars Under Drought Stress and Evaluation of Drought Resistance. North. Hortic..

[40] Yang N., Zhang Z.T., Zhang J.R., Yang X.F., Liu H., Chen J.Y., Shi L.S. (2025). Accurate estimation of winter-wheat leaf water content using continuous wavelet transform-based hyperspectral combined with thermal infrared on a UAV platform. Eur. J. Agron..

[41] Hang X.Z., Weng S.F., Yuan Z. (2014). Relationships between leaf traits of 5 plantscape shrubs and their responses to the environment in southern China. J. Northwest For. Univ..

[42] Galmés J., Ochogavía J.M., Gago J., Roldán E.J., Cifre J., Conesa M.À. (2013). Leaf responses to drought stress in Mediterranean accessions of Solanum lycopersicum: Anatomical adaptations to gas exchange parameters. Plant Cell Environ..

[43] Tian J.X., Wei L.P., He N.P., Xu L., Chen Z., Hou J.H. (2018). Vertical variation of leaf functional traits in temperate forest canopies in China. Acta Ecol. Sin..

[44] Li L., Ding J., Tian Y.Y., Yang H.Y., Feng J.C., Shi S. (2025). Functional traits and environmental adaptive characteristics of Ammopiptanthus mongolicus. Guihaia.

[45] Wang Z.Q., Huang H., Wang H., Peñuelas J., Sardans J., Niinemets Ü., Wright I.J. (2022). Leaf water content contributes to global leaf trait relationships. Nat. Commun..

[46] Gong H.D., Gao J. (2019). Soil and climatic drivers of plant SLA (specific leaf area). Glob. Ecol. Conserv..

[47] Zhu J.Y., Yu Q., Liu Y.P., Qin G.M., Li J.H., Xu C.Y., He W.J. (2018). Response of plant functional traits and leaf economics spectrum to urban thermal environment. J. Beijing For. Univ..

[48] Morera O.F., Stokes S.M. (2016). Coefficient α as a Measure of Test Score Reliability: Review of 3 Popular Misconceptions. Am. J. Public Health.

[49] Yin X., Struik P.C., Romero P., Harbinson J., Evers J.B., van der Putten P.E., Vos J. (2009). Using combined measurements of gas exchange and chlorophyll fluorescence to estimate parameters of a biochemical C photosynthesis model: A critical appraisal and a new integrated approach applied to leaves in a wheat (*Triticum aestivum*) canopy. Plant Cell Environ..

[50] Li Y., Liu Y.P., Zhang K.F., Li J., Zhang D.L. (2018). Effect of soil water stress on photosynthetic CO_2_ uptake and transport of cucumber in a greenhouse. J. Irrig. Drain..

[51] Douthe C., Dreyer E., Brendel O., Warren C.R. (2012). Is mesophyll conductance to CO_2_ in leaves of three *Eucalyptus* species sensitive to short-term changes of irradiance under ambient as well as low O_2_?. Funct. Plant Biol..

[52] Hassiotou F., Ludwig M., Renton M., Veneklaas E.J., Evans J.R. (2009). Influence of leaf dry mass per area, CO_2_, and irradiance on mesophyll conductance in sclerophylls. J. Exp. Bot..

[53] Sun J.W., Guan D.X., Wu J.B., Jing Y.L., Yuan F.H., Wang A.Z., Jin C.J. (2015). Day and night respiration of three tree species in a temperate forest of northeastern China. iFor. Biogeosci. For..

[54] Wang W.J., Ma D.M., Cai J.J., Huang T., Ma Q.L., Zhao L.J., Zhang Y. (2021). Photosynthetic characteristics of alfalfa seedlings under salt stress based on the FvCB model. Chin. J. Eco-Agric..

[55] Farquhar G.D., von Caemmerer S., Berry J.A. (1980). A biochemical model of photosynthetic CO_2_ assimilation in leaves of C_3_ species. Planta.

[56] Genty B., Briantais J.M., Baker N.R. (1989). The relationship between the quantum yield of photosynthetic electron transport and quenching of chlorophyll fluorescence. Biochim. Biophys. Acta.

[57] Peisker M., Apel H. (2001). Inhibition by light of CO_2_ evolution from dark respiration: Comparison of two gas exchange methods. Photosynth. Res..

[58] Valentini R., Mugnozza G.S., De Angelis P., Matteucci G. (1995). Coupling water sources and carbon metabolism of natural vegetation at integrated time and space scales. Agric. For. Meteorol..

[59] Laisk A.K. (1977). Kinetics of Photosynthesis and Photorespiration in C_3_ Plants.

[60] Von Caemmerer S. (2000). Biochemical Models of Leaf Photosynthesis.

