# Supplementary material for: Interaction of Soil Texture and Irrigation Level Improves Mesophyll Conductance Estimation
Source: Plants (Basel). 2025 Dec 12;14(24):3784. doi: 10.3390/plants14243784 (PMC12736796; doi:10.3390/plants14243784)
Supplement: Supplementary file 1 [file plants-14-03784-s001.zip › plants-3989776-supplementary.pdf]

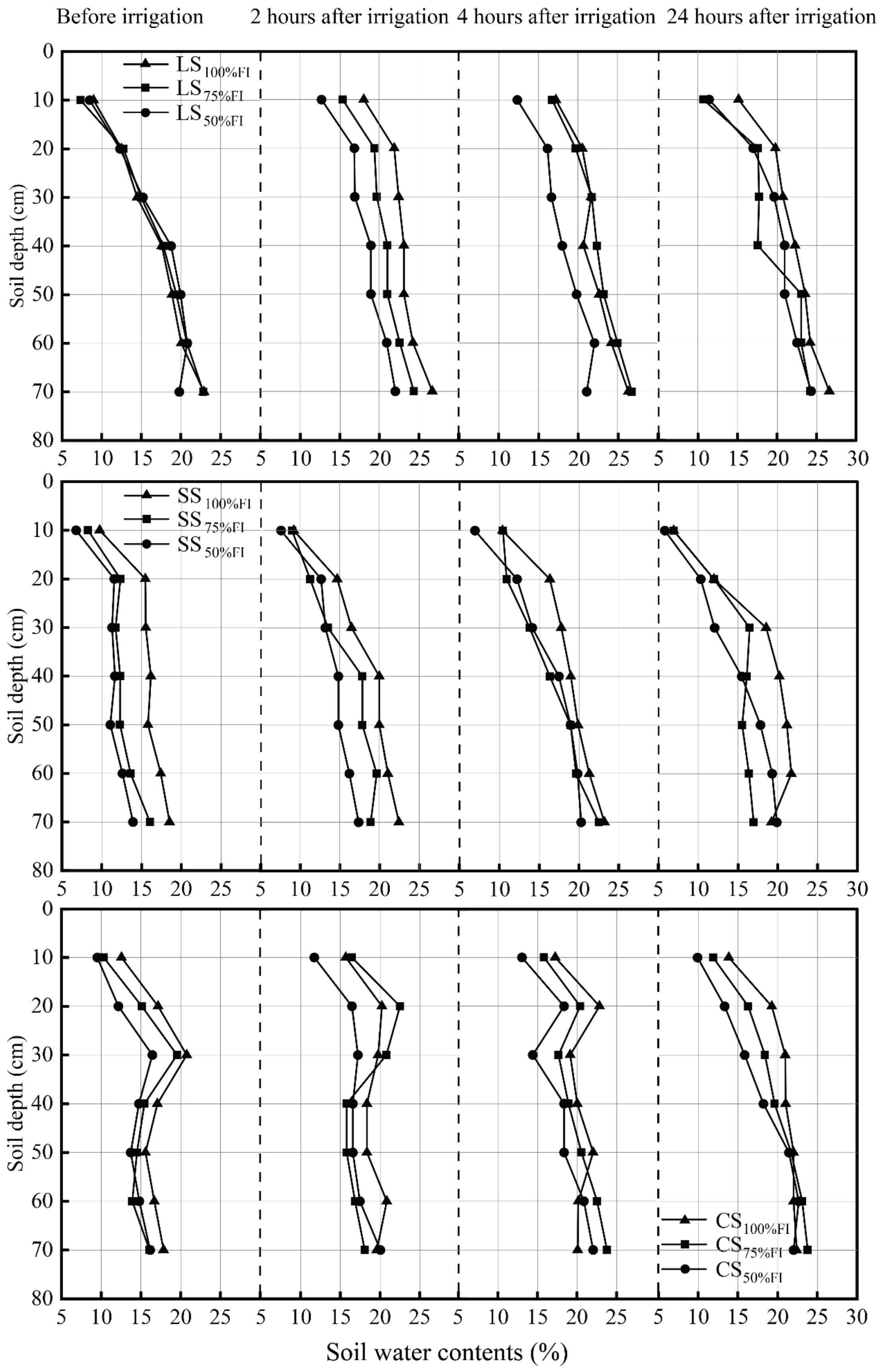


**Figure S1.** Differences in water diffusion and migration characteristics of different soil textures (LS (A), SS (B) and CS (C)) before irrigation and during different periods after irrigation.
